# Manganese transporter Slc39a14 deficiency revealed its key role in maintaining manganese homeostasis in mice

**DOI:** 10.1038/celldisc.2017.25

**Published:** 2017-07-18

**Authors:** Yongjuan Xin, Hong Gao, Jia Wang, Yuzhen Qiang, Mustapha Umar Imam, Yang Li, Jianyao Wang, Ruochen Zhang, Huizhen Zhang, Yingying Yu, Hao Wang, Haiyang Luo, Changhe Shi, Yuming Xu, Shintaro Hojyo, Toshiyuki Fukada, Junxia Min, Fudi Wang

**Affiliations:** 1 Department of Nutrition, Precision Nutrition Innovation Center, School of Public Health, Zhengzhou University, Zhengzhou, China; 2 Department of Nutrition, Nutrition Discovery Innovation Center, Institute of Nutrition and Food Safety, School of Public Health, The First Affiliated Hospital, Institute of Translational Medicine, School of Medicine, Collaborative Innovation Center for Diagnosis and Treatment of Infectious Diseases, Zhejiang University, Hangzhou, China; 3 Department of Neurology, The First Affiliated Hospital of Zhengzhou University, Zhengzhou University, Zhengzhou, China; 4 Osteoimmunology, Deutsches Rheuma-Forschungszentrum, Berlin, Germany; 5 Molecular and Cellular Physiology, Faculty of Pharmaceutical Sciences, Tokushima Bunri University, Tokushima, Japan; 6 Division of Pathology, Department of Oral Diagnostic Sciences, School of Dentistry, Showa University, Shinagawa, Japan; 7 RIKEN Center for Integrative Medical Sciences, Yokohama, Japan

**Keywords:** Slc39a14, Zip14, manganese, metal homeostasis, parkinsonism dystonia

## Abstract

SLC39A14 (also known as ZIP14), a member of the SLC39A transmembrane metal transporter family, has been reported to mediate the cellular uptake of iron and zinc. Recently, however, mutations in the *SLC39A14* gene have been linked to manganese (Mn) accumulation in the brain and childhood-onset parkinsonism dystonia. It has therefore been suggested that *SLC39A14* deficiency impairs hepatic Mn uptake and biliary excretion, resulting in the accumulation of Mn in the circulation and brain. To test this hypothesis, we generated and characterized global *Slc39a14*-knockout (*Slc39a14*^*−/−*^) mice and hepatocyte-specific *Slc39a14*-knockout (*Slc39a14*^*fl/fl*^*;Alb-Cre*^*+*^) mice. *Slc39a14*^*−/−*^ mice develop markedly increased Mn concentrations in the brain and several extrahepatic tissues, as well as motor deficits that can be rescued by treatment with the metal chelator Na_2_CaEDTA. In contrast, *Slc39a14*^*fl/fl*^*;Alb-Cre*^*+*^ mice do not accumulate Mn in the brain or other extrahepatic tissues and do not develop motor deficits, indicating that the loss of *Slc39a14* expression selectively in hepatocytes is not sufficient to cause Mn accumulation. Interestingly, *Slc39a14*^*fl/fl*^*;Alb-Cre*^*+*^ mice fed a high Mn diet have increased Mn levels in the serum, brain and pancreas, but not in the liver. Taken together, our results indicate that *Slc39a14*^*−/−*^ mice develop brain Mn accumulation and motor deficits that cannot be explained by a loss of *Slc39a14* expression in hepatocytes. These findings provide insight into the physiological role that SLC39A14 has in maintaining Mn homeostasis. Our tissue-specific *Slc39a14*-knockout mouse model can serve as a valuable tool for further dissecting the organ-specific role of SLC39A14 in regulating the body’s susceptibility to Mn toxicity.

## Introduction

In the body, metal homeostasis is a complex process involving multiple transporters that are tightly regulated by the levels of their substrates and/or other environmental conditions. The brain is particularly susceptible to the toxic effects of metals, and metals readily accumulate in the brain in the event of impaired metal homeostasis [1,2,3]. Manganese (Mn), an essential element for humans, is normally present in the brain, bone, liver, pancreas and kidney, and Mn imbalance has been linked to impaired brain functioning [ 4,5,6,7]. To date, several transporter proteins have been identified as having a role in maintaining Mn levels, including divalent metal ion transporter-1 (DMT1) [8,9,10,11], ferroportin (Fpn1) [12], SLC39A8 (also known as ZIP8) [13, 14], SLC30A10 (also known as ZnT10) [15,16,17] and SLC39A14 (also known as ZIP14) [4]. However, the precise molecular mechanisms that underlie Mn homeostasis are poorly understood.

The liver has a key role in maintaining Mn homeostasis in the body [18]. In patients, mutations in the hepatic metal ion transporter SLC39A8 have been linked to low blood Mn levels as well as several other human phenotypic traits. Recently, Lin *et al.* showed that both *Slc39a8*-knockout mice and mice in which *Slc39a8* expression is deleted specifically in the liver develop significantly decreased tissue Mn levels [13]. Conversely, SLC30A10 is a Mn exporter that replenishes serum Mn from tissue stores [19, 20]. Dietary Mn absorbed in the intestines is transported in the plasma by gamma globulin and albumin, although a small fraction of Mn is transported as a complex with transferrin and is taken up in neurons primarily via transferrin receptor 1 (TfR1, encoded in humans by the *TFRC* gene) [5, 21] and possibly other cells that express high levels of TfR1 [22]. In hepatocytes, Mn is largely sequestered in the mitochondria, where it performs numerous biological functions as a co-factor for many enzymes, including superoxide dismutase, arginase and glycosyltransferases [23, 24]. At the systemic level, Mn concentration is tightly regulated by the coordinated absorption and excretion of Mn [5]. Mn is excreted primarily via biliary and pancreatic secretion into the caudal sections of the gastrointestinal tract [25, 26], which help regulate the body’s Mn levels by adjusting the rate of elimination [27].

SLC39A14 was first discovered for its role in the uptake of zinc into the liver in response to acute inflammation and infection [28]. Mutations in *SLC39A14* can induce parkinsonism dystonia-like (PD-like) symptoms as a result of Mn accumulation in various brain structures, particularly the globus pallidus and striatum [4]. Similarly, mutations in other Mn transporters such as *SLC30A10* can also induce Mn neurotoxicity and its related symptoms [15, 29, 30]. However, despite the established role of Mn homeostasis in maintaining health, the tissue-specific *in vivo* role of Mn transporters has not been clearly defined.

Here, we report that global *Slc39a14*-knockout (*Slc39a14*^*−/−*^) mice and hepatocyte-specific *Slc39a14-*knockout (*Slc39a14*^*fl/fl*^*;Alb-Cre*^*+*^) mice have distinct Mn-related phenotypes and tissue metal distributions. Specifically, although *Slc39a14*^*−/−*^ mice recapitulate the hypermanganesemia that occurs in patients with PD-like syndrome [4], no Mn accumulation was observed in the brain or other tissues of *Slc39a14*^*fl/fl*^*;Alb-Cre*^*+*^ mice, which have considerably lower hepatic Mn levels. These findings provide new insight into the physiological role of SLC39A14 as a major Mn importer in the liver, and shed new light on the clinical effects of disrupting Mn homeostasis.

## Results

### *Slc39a14*^*−/−*^ mice develop progressive behavioral and motor deficits

**Figure 1:**
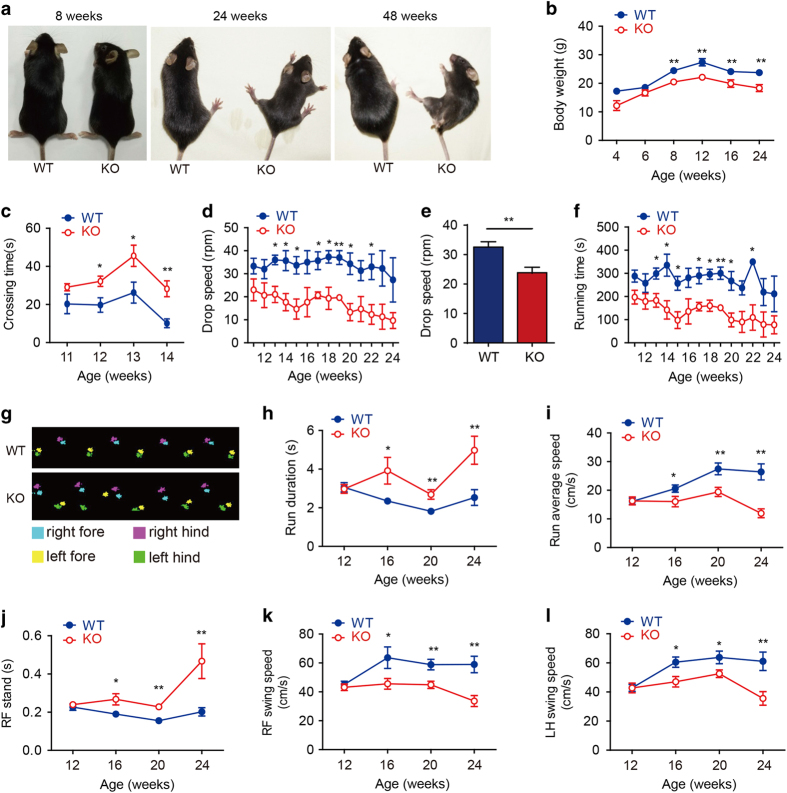
*Slc39a14*^*−/−*^ mice develop abnormal behavioral and motor phenotype. (**a**) Representative images of a wild-type (WT) and *Slc39a14*^*−/−*^ mouse (KO) at 8, 24 and 48 weeks of age. (**b**) Body weight of WT and KO littermates (*n*⩾4 mice/group). (**c**) Balance beam test results of WT and KO mice (*n*=3 mice/group). (**d**–**f**) Rotarod test results of WT and KO mice (*n*=3 mice/group). (**e**) The drop speed of WT and KO mice measured at 20 weeks of age (*n*=19 female mice and 20 male mice). (**g**) CatWalk analysis of a WT and KO mouse. Blue and purple represent the right forelimb (RF) and hindlimb (RH), respectively, and yellow and green represent the left forelimb (LF) and hindlimb (LH), respectively. (**h**–**l**) Analyses of CatWalk test results for WT and KO mice (*n*=3 mice/group). RF stands for right forelimb, LH stands for left hindlimb. *P<0.05 and **P<0.01, unpaired Student’s *t*-test.

Consistent with previous reports [31, 32], our homozygous *Slc39a14*-knockout (*Slc39a14*^*−/−*^) mice are viable and have no discernible morphological abnormalities at birth. However, by 4 weeks of age, these mice begin to show signs of progressive random torticollis and motor deficits (Figure 1a; Supplementary Movie S1, Supplementary Movie S2,Supplementary Movie S3,Supplementary Movie S4,Supplementary Movie S5). Compared with wild-type littermates, *Slc39a14*^*−/−*^ mice have lower body weight beginning at 8 weeks of age (Figure 1b). By six months of age, *Slc39a14*^*−/−*^ mice began to show signs of dystonia with a progressive inability to coordinate their motor activity (Figure 1c–i); these symptoms are similar to the PD-like motor disability in patients with a mutation in *SLC39A14* [4].

To further investigate the neurologically induced behavioral phenotypes in *Slc39a14*^*−/−*^ mice, we used the balance beam test [33, 34], the accelerating rotarod test [35, 36] and CatWalk gait analysis [37,38,39] to evaluate the animals’ walking ability and balance. In the balance beam test, the *Slc39a14*^*−/−*^ mice performed normally until 11 weeks of age, after which they began to show neurological deficits in terms of taking longer to cross the beam (Figure 1c). By 15 weeks of age, the *Slc39a14*^*−/−*^ mice were unable to complete the balance beam test, as they were unable to stay on to the beam. Similarly, in the rotarod test, the *Slc39a14*^*−/−*^ mice had significantly lower drop speed and running time compared with wild-type littermates; these deficits progressively worsened from 13 weeks of age to 24 weeks of age, the oldest age tested (Figure 1d–f). To assess gait and locomotion, we used the CatWalk test to visualize the animals’ footprints, and we calculated the print dimensions as well as the time and distance between footfalls. As shown in Figure 1g, the *Slc39a14*^*−/−*^ mice showed an irregular footprint patterns. The *Slc39a14*^*−/−*^ mice developed motor deficits beginning at 16 weeks of age, with significant differences in duration of running time (Figure 1h), average running speed (Figure 1i), time of right forelimb (RF) stand (Figure 1j), right forelimb (RF) and left hindlimb (LH) swing speed (Figure 1k and l) compared with wild-type littermates. Taken together, these results show that *Slc39a14*^*−/−*^ mice develop progressive motor deficits as early as 12 weeks of age.

### Loss of *Slc39a14* expression leads to Mn accumulation

**Figure 2:**
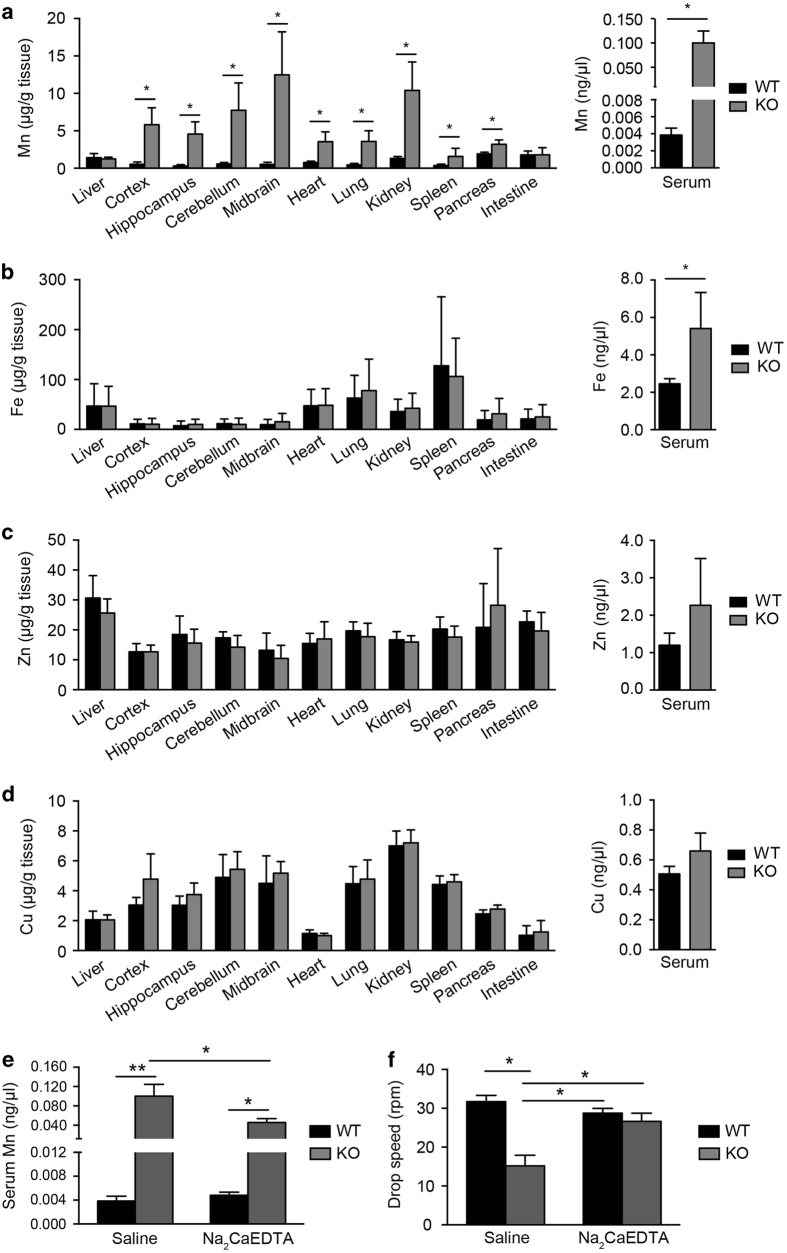
*Slc39a14*^*−/−*^ mice develop Mn accumulation in a variety of tissues. (**a**–**d**) Mn (**a**), Fe (**b**), Zn (**c**) and Cu (**d**) levels were measured in the indicated tissues of wild-type (WT) and *Slc39a14*^*−/−*^ (KO) mice at >24 weeks of age using ICP-MS (*n*=4 mice/group). In **a**, note the break in the *y*-axis for serum Mn. *P<0.05, unpaired Student’s *t*-test. (**e**) Serum Mn levels in *Slc39a14*^*−/−*^ mice treated for four weeks with Na_2_CaEDTA (370 mg/kg body weight) or saline (*n*=8–12 mice/group, 5 month of age). (**f**) Rotarod test results of *Slc39a14*^*−/−*^ mice treated for four weeks with Na_2_CaEDTA or saline (*n*=8–12 mice/group, 5 month of age). In **e** and **f**, *P<0.05 and **P<0.01, one-way ANOVA with Tukey’s *post hoc* test.

Slc39a14 has been reported to transport several metals, including Mn [4], zinc (Zn) [31] and iron (Fe) [40]. Therefore, we used inductively coupled plasma mass spectrometry (ICP-MS) to measure the levels of various metals in *Slc39a14*^*−/−*^ and wild-type mice [41,42,43]. Interestingly, compared with age-matched wild-type controls, at 48 weeks of age the *Slc39a14*^*−/−*^ mice had significantly higher levels of Mn in a wide range of tissues, including the serum, brain, kidney, lung, heart and spleen (Figure 2a); Mn levels in the liver and intestine were similar between wild-type and *Slc39a14*^*−/−*^ mice. We found no significant differences between wild-type and *Slc39a14*^*−/−*^ mice with respect to Fe, Zn or Cu in any tissues (Figure 2b–d), with the exception of a slight yet significant increase in serum Fe levels (Figure 2b). Taken together, these data indicate that Slc39a14 is predominantly a Mn transporter.

As early as 8 weeks of age, *Slc39a14*^*−/−*^ mice have brain Mn levels that are ~10-fold higher than age-matched wild-type control mice. We therefore tested whether the effects of Mn accumulation could be reduced by treating mice with the metal chelator Na_2_CaEDTA. At 5 months of age, we treated wild-type and *Slc39a14*^*−/−*^ mice with Na_2_CaEDTA for 4 weeks using a standard therapeutic protocol [44, 45]. Chelation therapy had no effect on serum Mn levels (Figure 2e) or rotarod performance (Figure 2f) in wild-type mice; however, in *Slc39a14*^*−/−*^ mice, Na_2_CaEDTA treatment significantly reduced serum Mn levels (from 0.1 ng μl^−1^ to 0.05 ng μl^−1^; Figure 2e) and significantly improved performance on the rotarod test (Figure 2f).

### Loss of *Slc39a14* expression alters the expression of various Mn transporters in several tissues

**Figure 3:**
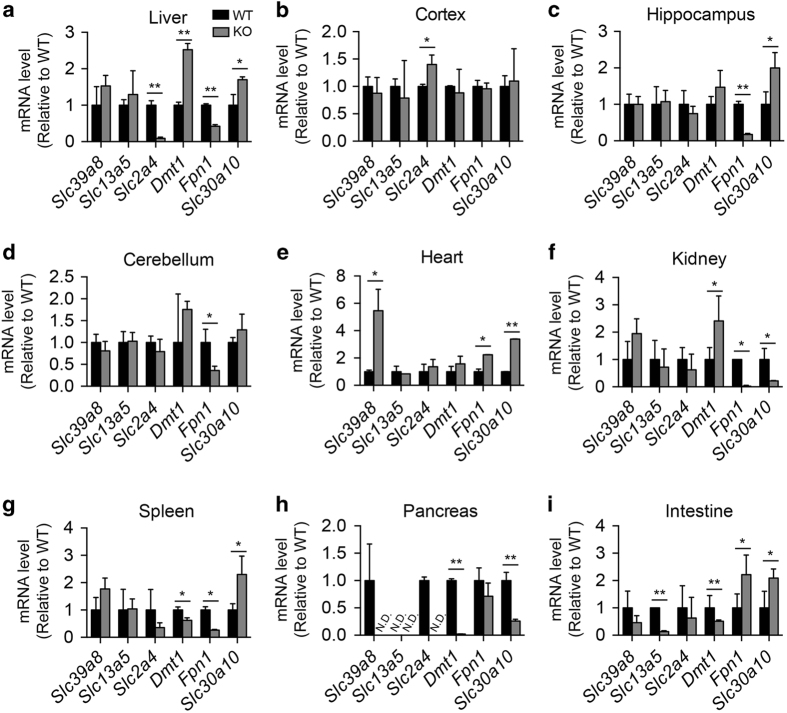
Expression levels of various Mn transporters in wild-type and *Slc39a14*^*−/−*^ mice. The mRNA levels of *Slc39a8*, *Slc13a5*, *Slc2a4*, *Dmt1*, *Fpn1* and *Slc30a10* were measured in the liver (**a**), cortex (**b**), hippocampus (**c**), cerebellum (**d**), heart (**e**), kidney (**f**), spleen (**g**), pancreas (**h**), intestine (**i**) of wild-type (WT) and *Slc39a14*^*−/−*^ (KO) mice at >24 weeks of age. (*n*=4 mice/group). N.D. stands for not detected, *P<0.05 and **P<0.01, unpaired Student’s *t*-test.

The expression of many metal transporters is highly dynamic and can be regulated by the concentration of their respective substrates [46]. In the absence of *Slc39a14* expression, other transporters (including Slc30a10, Fpn1, Dmt1, Slc39a8 and Mn citrate shuttle), glutamate receptors and calcium channels may have a compensatory role in maintaining normal hepatic Mn levels and intestinal Mn absorption [46]. We hypothesized that any differences in the expression of these transporters may contribute to the altered levels of Mn and the other metals observed in the *Slc39a14*^*−/−*^ mice. We therefore measured the effects of deleting *Slc39a14* expression on the expression of six genes that encode Mn transporters (*Slc39a8*, *Slc13a5*, *Slc2a4*, *Dmt1*, *Fpn1* and *Slc30a10*) in the liver, cortex, hippocampus, cerebellum, heart, kidney, spleen, pancreas and intestine (Figure 3a–i). We found that some genes are upregulated in some tissues and downregulated in other tissues, suggesting a highly dynamic regulatory process. Notably, the expression of Mn importers, including *Slc2a4*, was higher in the cortex of *Slc39a14*^*−/−*^ mice compared with wild-type mice (Figure 3b), whereas the Mn exporters *Slc30a10* and *Fpn1* were downregulated in the kidney. In the liver (Figure 3a), the expression of *Dmt1* and *Slc30a10* was significantly increased in the *Slc39a14*^*−/−*^ mice, whereas the expression of *Slc2a4* and *Fpn1* was significantly decreased. Nevertheless, this upregulation of Mn exporters (e.g., *Slc30a10,* Figure 3a,c,e,g and i) is not sufficient to prevent the accumulation of Mn due to the global loss of *Slc39a14* expression (Figure 2a).

### Hepatocyte-specific deletion of *Slc39a14* expression does not lead to Mn accumulation with standard dietary Mn

**Figure 4:**
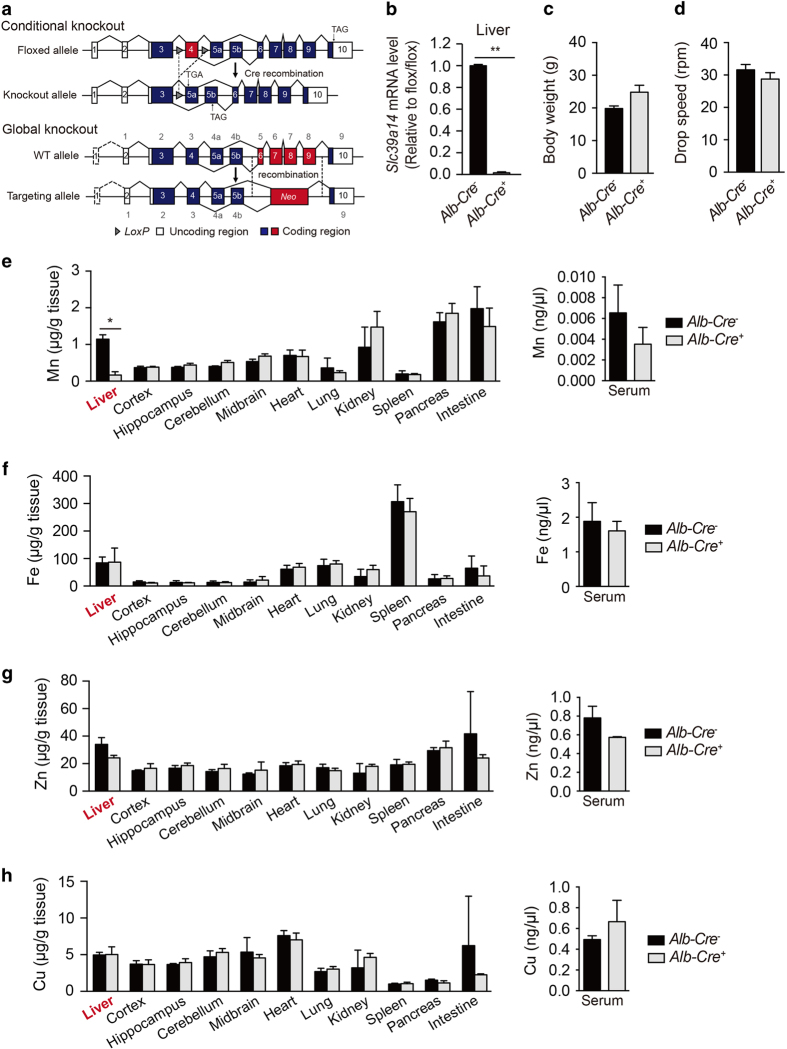
Hepatic Slc39a14 regulates Mn homeostasis by controlling the uptake of Mn in the liver. (**a**) Gene targeting strategy for generating the hepatocyte-specific *Slc39a14-*knockout mice (*Slc39a14*^*fl/fl*^*;Alb-Cre*^*+*^, upper panel) and *Slc39a14*^*−/−*^ mice (lower panel). (**b**) *Slc39a14* mRNA levels in the liver of *Slc39a14*^*fl/fl*^*;Alb-Cre*^*+*^ and control (*Slc39a14*^*fl/fl*^*;Alb-Cre*^*−*^) mice (*n*=4 mice/group). (**c**, **d**) Summary of body weight (**c**) and rotarod test results (**d**) for *Slc39a14*^*fl/fl*^*;Alb-Cre*^*+*^ and control mice (*n*=4 mice/group). (**e**–**h**) Levels of Mn (**e**), Fe (**f**), Zn (**g**) and Cu (**h**) were measured in the indicated tissues of *Slc39a14*^*fl/fl*^*;Alb-Cre*^*+*^ and control mice at 8–12 weeks of age (*n*=3 mice/group). *P<0.05 and **P<0.01, unpaired Student’s *t*-test.

*Slc39a14* is highly expressed in hepatocytes [47]. Moreover, mutations in the human *SLC39A14* gene have been suggested to affect Mn transport primarily in hepatocytes, thereby underlying the parkinsonism dystonia seen in patients with such mutations; data obtained with a transgenic zebrafish model appear to support this notion [4]. To test directly whether a loss of Slc39a14 selectively in hepatocytes affects hepatic Mn uptake and causes Mn accumulation in the blood and brain, we generated hepatocyte-specific *Slc39a14*-knockout mice (*Slc39a14*^*fl/fl*^*;Alb-Cre*^*+*^ mice) using a conditional knockout strategy (Figure 4a). The murine *Slc39a14* gene encodes two isoforms that contain either exon 5a or 5b [32]. We therefore targeted exon 4, which introduces a premature stop codon in both isoforms (Figure 4a). qPCR analysis confirmed that hepatic expression of *Slc39a14* was virtually abolished in *Slc39a14*^*fl/fl*^*;Alb-Cre*^*+*^ mice compared with control mice (Figure 4b).

Unlike *Slc39a14*^*−/−*^ mice, the hepatocyte-specific *Slc39a14*-knockout mice do not develop torticollis (data not shown), reduced body weight (Figure 4c), or neurological deficits (Figure 4d), suggesting that dysregulated Mn metabolism in non-hepatic tissues contributes to the phenotype in *Slc39a14*^*−/−*^ mice. Moreover, consistent with the targeted deletion of *Slc39a14* expression in the liver, *Slc39a14*^*fl/fl*^*;Alb-Cre*^*+*^ mice had normal levels of Mn, Zn and Cu in all tissues examined (Figure 4e–h), with the notable exception of significantly reduced hepatic Mn levels (Figure 4e). Thus, deleting *Slc39a14* expression selectively in hepatocytes does not induce systemic Mn accumulation, supporting the notion that the liver is the primary reservoir for Slc39a14-mediated Mn uptake in mice.

### High dietary Mn does not lead to hepatic Mn accumulation in hepatocyte-specific *Slc39a14*-knockout mice

**Figure 5:**
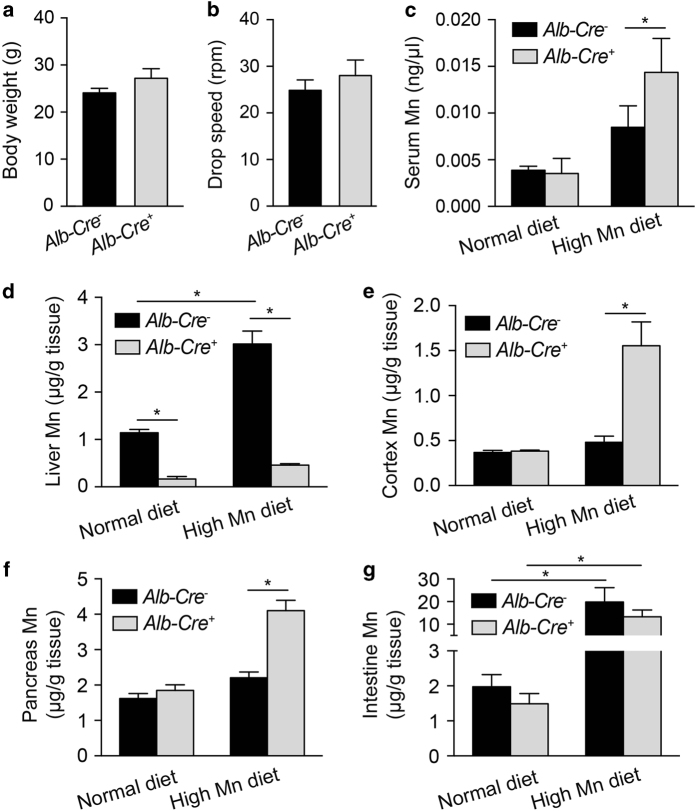
Summary of tissue Mn levels in hepatocyte-specific *Slc39a14*-knockout mice. *Slc39a14*^*fl/fl*^*;Alb-Cre*^*+*^ and control mice were fed a high Mn diet for 4 weeks. (**a**, **b**) Summary of body weight (**a**) and rotarod test results (**b**) for *Slc39a14*^*fl/fl*^*;Alb-Cre*^*+*^ and control mice after consuming a high Mn diet for 4 weeks (*n*=4 mice/group,5 month of age). (**c**–**g**) Mn levels were measured in the serum, liver, cortex, pancreas and intestine of *Slc39a14*^*fl/fl*^*;Alb-Cre*^*+*^ and control mice after 4 weeks on a diet containing normal Mn or high Mn (*n*=4–6 mice/group, 5 month of age). In **c**–**g**, *P<*0.05*, one-way ANOVA with Tukey’s *post hoc* test.

Given the systemic accumulation of Mn in *Slc39a14*^*−/−*^ mice, and that the liver is the primary storage site for Slc39a14-mediated Mn uptake in mice, we hypothesized that feeding hepatocyte-specific *Slc39a14*^*fl/fl*^*;Alb-Cre*^*+*^ mice a high Mn diet might increase their brain Mn levels. We therefore fed newly weaned *Slc39a14*^*fl/fl*^*;Alb-Cre*^*+*^ and control (*Slc39a14*^*fl/fl*^*;Alb-Cre*^*−*^) mice a purified AIN-93G diet containing either a normal (10 p.p.m.) or high (2 400 p.p.m.) concentration of Mn [48]. After 30 days on a high Mn diet, neither body weight (Figure 5a) nor rotarod performance (Figure 5b) differed between *Slc39a14*^*fl/fl*^*;Alb-Cre*^*+*^ and control mice; we also found no difference between mice fed a normal Mn diet and mice fed a high Mn diet (compare Figure 5a and b with Figure 4c and d, respectively). However, the high Mn diet increased serum Mn levels in both *Slc39a14*^*fl/fl*^*;Alb-Cre*^*+*^ and control mice, with a much stronger effect on the *Slc39a14*^*fl/fl*^*;Alb-Cre*^*+*^ mice (Figure 5c).

Interestingly, and consistent with the selective loss of *Slc39a14* expression in the liver, hepatic Mn levels remained extremely low in the high Mn-fed *Slc39a14*^*fl/fl*^*;Alb-Cre*^*+*^ mice; in these mice, hepatic Mn levels were even lower than in control mice fed a normal Mn diet (Figure 5d). In contrast, Mn levels were significantly higher in the brain (Figure 5e) and pancreas (Figure 5f) of high Mn-fed *Slc39a14*^*fl/fl*^*;Alb-Cre*^*+*^ mice compared with both control mice and *Slc39a14*^*fl/fl*^*;Alb-Cre*^*+*^ mice fed a normal Mn diet; intestinal Mn levels were significantly increased in both *Slc39a14*^*fl/fl*^*;Alb-Cre*^*+*^ and control mice, but did not differ significantly between genotypes (Figure 5g). Finally, ICP-MS measurements showed that feeding *Slc39a14*^*fl/fl*^*;Alb-Cre*^*+*^ mice a high Mn diet had no significant effect on Fe, Zn or Cu levels in any tissues examined.

## Discussion

Here, we characterized the role of the metal ion transporter Slc39a14 in systemic Mn metabolism using both global *Slc39a14-*knockout and hepatocyte-specific *Slc39a14*-knockout mice. Our results reveal that hepatic Slc39a14 has a specific role in maintaining systemic Mn homeostasis. Specifically, we found that Slc39a14 regulates Mn uptake primarily in the liver, which in turn mediates Mn levels in other tissues, including the brain, heart, kidney and circulation. Moreover, our results indicate that Slc39a14 functions to control the influx of Mn in the liver, as *Slc39a14*^*−/−*^ mice develop Mn accumulation in the brain and other tissues, but not in the liver. Furthermore, we found that hepatocyte-specific *Slc39a14*-knockout mice do not develop signs of Mn accumulation under normal dietary Mn conditions; however, upon consuming a high Mn diet, these mice develop increased Mn levels in the brain and serum, but not in the liver. Taken together, these findings indicate that hepatic Slc39a14 regulates Mn homeostasis by mediating the hepatic uptake of Mn, providing insight into the function of SLC39A14 in humans.

Maintaining Mn homeostasis is essential for human health, and excess Mn has been linked to brain Mn accumulation and neurodegeneration [49, 50]. Mutations in the Mn transporters SLC39A14 and SLC30A10 have been associated with increased Mn levels in the serum and brain [4, 30] and the development of PD-like symptoms. Moreover, exposure to occupational and other environmental sources of high Mn can cause similar neurological symptoms [50, 51]. The patients with a homozygous mutation in *SLC39A14* have increased levels of Mn in the blood and brain at an early age and develop dystonia-parkinsonism [4]. However, unlike patients with a mutation in *SLC30A10*, these patients do not have increased hepatic Mn levels, nor do they develop liver disease. In addition, Tuschl *et al.* deleted *Slc39a14* expression in zebrafish and found increased Mn levels in the brain, but not in the abdominal viscera. In contrast, our experiments using mouse knockout models provide a causal link between SLC39A14 function, hepatic Mn import and Mn-related neurotoxicity. Unfortunately, performing these experiments in humans would not be possible.

A key finding of our study is that SLC39A14 has a central role in regulating hepatic Mn uptake. We found that selectively deleting *Slc39a14* expression in hepatocytes does not cause Mn overload in the brain, blood or other tissues. In contrast, global *Slc39a14*-knockout mice have dysregulated Mn metabolism and develop behavioral and motor deficits similar to the movement disorder symptoms in patients with an *SLC39A14* mutation [4]. Our analysis of hepatocyte-specific *Slc39a14*-knockout mice indicates that the accumulation of Mn in the blood and brain does not arise solely from impaired hepatic Mn uptake. Although *Slc39a14*^−/−^ mice were previously reported to have reduced zinc uptake and increased iron absorption [31], our ICP-MS data show that *Slc39a14*^*−/−*^ mice have normal tissue levels of Fe, Zn and Cu (with the sole exception of increased serum Fe levels). Taken together, our data provide compelling evidence that globally deleting *Slc39a14* expression primarily affects Mn metabolism, but has little effect on Fe, Zn or Cu metabolism, thereby indicating that Slc39a14 functions primarily as a Mn transporter.

The mechanism underlying Mn toxicity is poorly understood. Although the central nervous system is a well-known primary target for Mn, how Mn is transported across the blood–brain barrier is currently unknown. Based on our results, we believe that SLC39A14 does not directly mediate the uptake of Mn into the brain; rather, hepatic SLC39A14 has systemic effects on Mn homeostasis. In humans, dietary Mn is delivered to the liver, where it forms a conjugate with bile and is then excreted into the intestine [52, 53]. In mammals, Mn is excreted into the caudal parts of the intestinal tract [26]. Given that Slc39a14 is expressed on the basolateral membrane of mucosal cells in the proximal intestine [54], it is possible that Slc39a14 has a role in excreting Mn from the proximal intestine. In addition, our data suggest that the differences in susceptibility to Mn accumulation between different tissues may be attributed to different tissue distributions of the major Mn transporters.

Mn toxicity has been linked directly to behavioral and movement disorders in both humans and animal models [51, 55], and Na_2_CaEDTA is often used to chelate high levels of heavy metals, albeit with mixed results. For example, Na_2_CaEDTA chelation therapy reduced Mn load and reversed Mn-related neurotoxicity in one patient with an *SLC39A14* mutation, but had no effect in another patient with the same mutation [4]. We found that *Slc39a14*-knockout mice develop behavioral and motor deficits associated with high levels of Mn in the brain, and Na_2_CaEDTA treatment significantly decreased serum Mn content and improved locomotor function in these mice.

**Figure 6:**
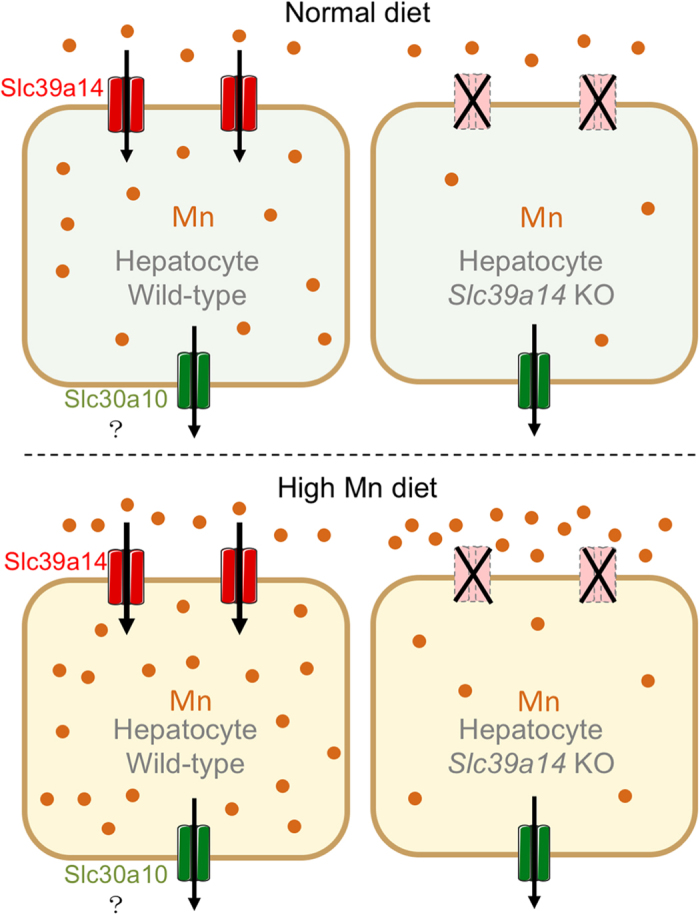
Schematic model depicting the role of hepatic Slc39a14 on intracellular and systemic Mn levels. (Top) Under normal dietary Mn conditions with normal Slc39a14 levels (left), Mn is absorbed by hepatocytes via the Mn transporter Slc39a14 (shown in red) and may be exported via the Mn exporter Slc30a10 (shown in green). In contrast, loss of *Slc39a14* expression leads to significantly decreased Mn levels in hepatocytes (right). (Bottom) Consuming a high Mn diet fails to overcome the reduced Mn levels in *Slc39a14*-knockout hepatocytes, leading to increased systemic Mn accumulation.

Based on our findings, we suggest a model in which hepatic SLC39A14 has a role in maintaining systemic Mn homeostasis (Figure 6). In this model, under normal dietary Mn, Mn is absorbed into wild-type hepatocytes via Slc39a14. If *Slc39a14* expression is deleted in hepatocytes, their intracellular Mn levels are reduced considerably. Under high dietary Mn, intracellular Mn increases considerably in wild-type hepatocytes, but not in *Slc39a14*-deficient hepatocytes, regardless of extracellular Mn concentration. At the systemic level, deleting *Slc39a14* expression leads to Mn accumulation in the brain and other tissues, but not in the liver, suggesting that the brain in *Slc39a14*^*−/−*^ mice is highly susceptible to Mn toxicity. On the other hand, hepatocyte-specific *Slc39a14*-knockout mice do not develop Mn accumulation in the brain, blood or other tissues; if challenged with a high Mn diet, however, these mice develop increased Mn levels in the brain, possibly due to the inability to sequester Mn in the liver. Although further studies are needed in order to determine the tissue-specific role that SLC39A14 has in Mn metabolism, our mouse models provide a valuable tool for investigating the underlying mechanisms by which Mn transporters regulate Mn homeostasis and for testing new pharmaceutical approaches to manage Mn toxicity in neurodegenerative diseases.

## Materials and Methods

### Animals

All mice were housed in a specific pathogen-free facility and maintained on a purified AIN-76A diet (Research Diets, New Brunswick, NJ, USA). All mice were maintained under a 12-hour light/dark cycle, and all animal experiments were approved by the Institutional Animal Care and Use Committee of Zhengzhou University.

Heterozygous *Slc39a14*^*+/−*^ mice [32] on a mixed background were backcrossed to C57BL6/J mice more than five generations. *Slc39a14*^*+/−*^ mice were then crossed to generate litters containing *Slc39a14*^*+/+*^, *Slc39a14*^*+/−*^ and *Slc39a14*^*−/−*^ mice. *Slc39a14*^*fl/fl*^*;Alb-Cre*^*+*^ mice were generated by gene targeting ES cells in order to delete exon 4 in the *Slc39a14* gene, resulting in *Slc39a14*^*fl/+*^ mice (Shanghai Biomodel Organism, Shanghai, China). *Slc39a14*^*fl/+*^ mice were backcrossed to C57BL6/J mice more than seven generations, and *Slc39a14*^*fl/fl*^ mice were bred with *Alb-Cre* transgenic mice to generate *Slc39a14*^*fl/fl*^*;Alb-Cre*^*+*^ and *Slc39a14*^*fl/fl*^*;Alb-Cre*^*−*^ offspring.

### Animal genotyping

Genomic DNA was extracted from mouse tail biopsies using the TIANamp Genomic DNA Kit (Tiangen Biotech, Beijing, China; Cat. #DP304). The following primer pair was used to genotype the *Slc39a14*^*fl/fl*^ mice: CTGTGGTCTTCCTGCCTTGG and TACCCTGCCCTACACGACTC; the following primer pair was used to genotype the *Slc39a14*^*fl/fl*^*;Alb-Cre* mice: GCAAACATACGCAAGGGATT and AGGCAAATTTTGGTGTACGG. To genotype the offspring from the *Slc39a14*^*+/−*^ crosses, two forward primers ( TGCTGCTGCTATTTGGGTCT or CTCGTGCTTTACGGTATCGC to amplify the wild-type and mutant alleles, respectively) were used with a single reverse primer ( GAATGCTGCATTGAAAAGGTC).

### Behavioral testing

To phenotype the mice in this study, we used the balance beam, accelerating rotarod and CatWalk tests. For each experiment, four pairs of knockout mice and control littermates were used. The mice were trained for three consecutive days and were tested on the fourth day. Thereafter, the tests were repeated once a week until the mice could no longer complete the tests.

For the balance beam [33, 34], a 100-cm long, 10-mm thick beam was placed at a height of 70 cm with an enclosed goal box at one end. During training, the mouse was allowed to acclimate to the goal box for 10 min. The mouse was then trained to walk the beam from the starting point to the goal box. On the testing day, the time taken to travel from the starting point to the goal box was recorded.

The accelerating rotarod [35, 36] (Softmaze-XR1514, Shanghai, China) consisted of a felt-covered steel cylinder fitted to a rotating pump with a variable speed setting. Age-matched animals were trained on the rotarod for three consecutive days, with three 5-min training trials with the speed set to 10, 12 and 14 r.p.m. After these three training days, the mice were tested. During testing, the rotarod speed was increased from 4 to 40 r.p.m. over a 5-min period, and both the speed at which the mouse fell off the rod (in r.p.m.) and total running time (in seconds) were measured.

To analyze the animals’ gait, we used the CatWalk XT system [37–39] (Noldus Information Technology, Wageningen, The Netherlands). To measure the gait, the mouse was placed on a glass plate and allowed to walk along a narrow walkway, with a green light on the walkway and a red ceiling light to illuminate the mouse; a camera was used to record the mouse’s paw placement while walking. During the test, the mouse was placed in an enclosed box at the end of the walkway and allowed to acclimate for 10 min, after which the lights were turned off, and the mouse was allowed to walk from the beginning of the walkway to the end of the walkway and enter the box. A successful trial was defined as the mouse taking ⩽10 s to cross the field of vision of a video camera that was used to capture the mouse’s movement, which was analyzed by CatWalk XT software based on the mouse’s limbs. Each mouse successfully completed three trials on each training day and two trials on the test day.

### Laboratory measurements for ICP-MS detection

These methods were performed as previously described [4, 41,42,43]. Approximately 10–200 mg of each organ was placed in a 25-ml PFA vial with a screw cap and digested with 3.4 ml of concentrated EMSURE nitric acid solution (65%, 1.39 g ml^−1^, Merck, Darmstadt, Germany). The following microwave program was used for digestion: the temperature of the sample was ramped from room temperature to 200 degrees by microwaving at 1 000 W for 20 min; the microwave power was then held at 1 000 W for an additional 30 min, followed by a 15-min cooling step. The total digestion time was therefore 65 min. The digested samples were dried by heating and immediately diluted to a final volume of 5 ml in high-purity deionized water (18 MΩ cm^−1^ resistivity) obtained from a Milli-Q Integral 10 purification system (EMD Millipore Corporation, Darmstadt, Germany). Multi-element standard solutions containing Mn, Fe, Zn and Cu were prepared by diluting and mixing individual element standard stock solutions (1 000 μg ml^−1^) obtained from the National Institute of Quality Standards (Beijing, China). An Agilent 7700x ICP-MS equipped with an Agilent ASX 520 auto-sampler was used to measure elements.

### RNA extraction and qPCR analysis

Total RNA was extracted from the mouse tissues using TRIzol reagent (Invitrogen, Shanghai, China), and cDNA was synthesized using the PrimerScript RT reagent Kit with gDNA Eraser (Takara, Beijing, China, cat. #RR047). Real-time PCR was performed using the two-step quantitative RT-PCR method in accordance with the manufacturer’s instructions (Takara, cat. #RR820) on a QuantStudio 7 Flex Real-Time PCR System (ThermoFisher Applied Biosystems, Waltham, MA, USA). The expression level of each target gene was normalized to the sample’s *Gapdh* mRNA level and is expressed relative to the respective wild-type level. The following primer sets were used:

*Slc39a14* forward 5′- TTTCCCAGCCCAAGGAAG-3′ and reverse 5′- CAAAGAGGTCTCCAGAGCTAAA-3′; *Dmt1* forward 5′- CGGGGATGAATGACTTCCTG-3′ and reverse 5′- GGACATAAACCACTACAAAGTACA-3′; *Slc39a8* forward 5′- TGCCTGGATGATCACGCTTT-3′ and reverse 5′- CGGGTGCTCATTCCTGCAT-3′; *Slc30a10* forward 5′- GCCACCTTGCACATCAAACA-3′ and reverse 5′- GCTTCTTAGCGCAGCTCTGG-3′; *Slc13a5* forward 5′- CAGGGCTCTCGAAGTGGATG-3′ and reverse 5′- GAATCATGACATACAGAGGATGGA-3′; *Slc2a4* forward 5′- AACGGGTTCCAGCCATGAG-3′ and reverse 5′- AACCCATGCCGACAATGAAG-3′; *Fpn1* forward 5′- GTCGGCCAGATTATGACATTTG-3′ and reverse 5′- ATTCCAACCGGAAATAAAACCA-3′.

### Na_2_CaEDTA chelation therapy of *Slc39a14*^*−/−*^ mice

*Slc39a14*^*−/−*^ mice received a daily i.p. injection of 370 mg kg^−1^ Na_2_CaEDTA (disodium calcium edetate, also known as edetate calcium disodium, ethylene diamine tetra-acetic acid disodium calcium salt) [44, 45] for four days, followed by three days without injection. After four weeks of this weekly treatment cycle, performance on the rotarod test was measured.

### Dietary Mn supplementation in hepatocyte-specific *Slc39a14*-knockout mice

Control (*Slc39a14*^*fl/fl*^*;Alb-Cre*^*−*^) and *Slc39a14*^*fl/fl*^*;Alb-Cre*^*+*^ mice were fed a purified AIN-93G diet containing either normal (10 p.p.m.) Mn (diet D08080401) or high (2 400 p.p.m.) Mn (diet D17020702) for 30 days. Based on a previous study, 2 400 p.p.m. of dietary Mn does not induce toxicity [48]. During the 30-day feeding period, body weight, motor performance and ICP-MS metal analysis were performed as described above.

### Statistical analysis

At least three independent experiments were used for statistical analyses. GraphPad Prism 5 software was used for statistical analyses, and all data passed the test for normality. Multiples groups were analyzed using a one-way ANOVA with Tukey’s *post hoc* test, and differences between two groups were analyzed using the Student’s *t*-test. Differences with a *P*-value <0.05 were considered significant. All summary data are presented as the mean±s.e.m.
