# Supplementary material for: Manganese transporter Slc39a14 deficiency revealed its key role in maintaining manganese homeostasis in mice
Source: Cell Discov. 2017 Jul 18;3:17025–. doi: 10.1038/celldisc.2017.25 (PMC5519003; doi:10.1038/celldisc.2017.25)
Supplement: Supplementary Information [file celldisc201725-s1.pdf]

Supplemental Files

Manganese transporter Slc39a14 deficiency revealed its key role in maintaining manganese homeostasis in mice

Xin et al.

Supplemental Movie S1. Rotarod testing of a male *Slc39a14*$^{-/-}$ mouse (KO) and a male wild-type mouse.

Supplemental Movie S2. Rotarod testing of a female *Slc39a14*$^{-/-}$ mouse (KO) and a female wild-type mouse.

Supplemental Movie S3. Tail suspension test of an *Slc39a14*$^{-/-}$ mouse (KO) and a wild-type mouse.

Supplemental Movie S4. An *Slc39a14*$^{-/-}$ mouse (KO), showing reduced exploratory activities on the cover of the cage compared to a wild-type littermate.

Supplemental Movie S5. An *Slc39a14*$^{-/-}$ mouse (KO), showing reduced exploratory activities on the bottom of the cage compared to a wild-type littermate.
